# Depression does not affect time perception and time-to-contact estimation

**DOI:** 10.3389/fpsyg.2014.00810

**Published:** 2014-07-24

**Authors:** Daniel Oberfeld, Sven Thönes, Benyne J. Palayoor, Heiko Hecht

**Affiliations:** Department of Psychology, Section Experimental Psychology, Johannes Gutenberg-Universität MainzMainz, Germany

**Keywords:** depression, time perception, timed action task, internal clock, verbal time estimation, time production, time reproduction, time-to-contact estimation

## Abstract

Depressed patients frequently report a subjective slowing of the passage of time. However, experimental demonstrations of altered time perception in depressed patients are not conclusive. We added a timed action task (time-to-contact estimation, TTC) and compared this indirect time perception task to the more direct classical methods of verbal time estimation, time production, and time reproduction. In the TTC estimation task, the deviations of the estimates from the veridical values (relative errors) revealed no differences between depressed patients (*N*= 22) and healthy controls (*N*= 22). Neither did the relative errors of the TTC estimates differ between groups. There was a weak trend toward higher variability of the estimates in depressed patients but only at the shortest TTC and at the fastest velocities. Time experience (subjective flow of time) as well as time perception in terms of interval timing (verbal estimation, time production, time reproduction) performed on the same subjects likewise failed to produce effects of depression. We conclude that the notion that depression has a sizeable effect on time perception cannot be maintained.

## INTRODUCTION

Depressed patients frequently report a slowing and sometimes even an apparent arrest of the passage of time (e.g., Straus, 1947; Dubois, 1954; Grondin et al., 2006; Gil and Droit-Volet, 2009), using statements like “Every hour seems a year to me” (Mezey and Cohen, 1961) or “Time doesn’t seem to move at all” (Straus, 1947). Empirical studies provide evidence that the passing of time can be subjectively slower in depressed patients than in healthy controls (e.g., Mezey and Cohen, 1961; Bech, 1975; Wyrick and Wyrick, 1977; Richter and Benzenhöfer, 1985; Münzel et al., 1988). On the basis of these results on the subjective passage of time in depressive patients, more recent studies investigated potential effects of depression on time perception by means of different laboratory tasks that are state-of-the-art in research on time perception (see Grondin, 2010 for a recent review; see also Kuhs et al., 1989; Blewett, 1992; Sévigny et al., 2003; Bschor et al., 2004; Mahlberg et al., 2008; Msetfi et al., 2012). Basically, three tasks have been used; (a) *verbal time estimation*, where the subject is asked to give an estimate in time units like seconds or minutes of a presented time interval, which is marked for instance by two brief tones or comparable visual stimuli for example flashes (e.g., Dilling and Rabin, 1967; Bech, 1975; Kitamura and Kumar, 1983; Bschor et al., 2004), (b) *time production,* where a time interval is specified in temporal units and the subject is asked to produce this interval for example by pressing a button to mark the interval’s beginning and end (e.g., Tysk, 1984; Münzel et al., 1988) and (c) *time reproduction,* where a time interval is presented first as in (a) and in a second step the subject reproduces the interval as in (b) based on his (short term) memory representation of the interval (Mundt et al., 1998; Mahlberg et al., 2008).

These three tasks have in common that they measure the estimation or production of defined time intervals rather than a general subjective experience of the flow of time. Still, all of the three tasks focus on conscious experience of time. However, they differ in other aspects that make comparisons across tasks difficult. For instance, the role of motor activity clearly differs between the tasks. Time production and reproduction require timed motor responses. In contrast, time estimation, and of course judgments on the flow of time, do not. This is crucial in the context of investigating depressive samples because a well-known concomitant feature of depression is psychomotor retardation (e.g., Lemke et al., 1999; Buyukdura et al., 2011; Calugi et al., 2011). Therefore, production and reproduction especially of short intervals might be influenced by motor-related impairments in depressive subjects whereas verbal estimation could provide data that are independent of this variable.

Moreover, depending on the task, it has to be considered that memory processes are involved differently. Time production and verbal estimation require the subject to retrieve long term memory samples of the intervals to be produced or estimated. Time reproduction, in contrast, requires subjects to rehearse the representation of an interval, which has been presented within a given trial or experimental block, in working memory. Thus, time reproduction is rather demanding on short-term memory or sensory memory.

Despite the large number of studies, a firm conclusion to the effect that depression does indeed alter time perception cannot be drawn. Not only are the results mixed at best (Msetfi et al., 2012), more importantly, the use of the classical time estimation tasks is rather removed from everyday timed action. We suggest to add a timed action task to the repertoire of traditional timing tasks, which does not require conscious focus on time, in the sense of explicit judgments in time units or productions of a time interval. The time-to-contact (TTC) estimation paradigm (for an overview see Hecht and Savelsbergh, 2004) is particularly suited for the job as it works with short durations that have typically been used in the classical time perception tasks with depressed patients. TTC is defined as the time a moving object will take to arrive at a designated position in space. TTC judgments are important in everyday situations like crossing a street (“Will I have enough time to cross before the approaching car reaches my position?”), or catching a ball (“When will I have to start closing my hand?”; cf. Regan and Gray, 2000). Absolute TTC estimates can be obtained by means of a so-called prediction-motion (PM) task (e.g., Schiff and Detwiler, 1979). In this task, a moving object approaches the observer (or another object) on a collision course. Some time before the object reaches the observer, it is occluded from view. The task of the observer is to press a button at exactly the moment when the object would have arrived at his or her position. It has been suggested that this task comprises a timing component (cf. Tresilian, 1995). Observers in a PM task might first estimate TTC at the moment when the object disappears from their view, and then use a timing mechanism to delay their response until the anticipated collision time (Baurès et al., 2011; but see DeLucia and Liddell, 1998). This would approximately correspond to the time production task, with the important difference that for TTC estimation the to-be-timed interval is not specified in time units but needs to be estimated from visual parameters. We have conducted an experiment in which we compared performance on a TTC estimation task to performance on the traditional tasks. Before reporting the experiment, we first address the background of the major traditional timing tasks, in particular the model of an internal clock.

### THE INTERNAL CLOCK HYPOTHESIS

Probably the most influential model of time perception is the *internal clock model* (e.g., Creelman, 1962; Treisman, 1963; Allan et al., 1974; Gibbon et al., 1984). According to this model, a pacemaker generates pulses, which are received by an accumulator (i.e., a “counter” in the broader sense). The more pulses it has accumulated, the longer the perceived interval. For judging the length of a time interval or for comparing two time intervals, a memory component and a decision device (“comparator”) are required. These different components, pacemaker, accumulator, memory stage, and comparator, are formalized in scalar expectancy theory (SET; Gibbon, 1977; Gibbon et al., 1984), originating from research on animal as well as human timing. The clock speed could be altered by factors like arousal (e.g., Tecce, 1972; Wearden and Pentonvoak, 1995; Mella et al., 2011) or neurological and psychiatric disorders (Allman and Meck, 2012). The internal clock model predicts that the time estimates obtained by the three classical methods are systematically related to the speed of the internal clock.

If the clock runs at a fast rate, then in the *production task,* the number of clock pulses corresponding subjectively to, e.g., “1 s” will be reached earlier than when the clock runs slower. Therefore, shorter intervals will be produced if the clock runs fast. In contrast, in the *verbal estimation task,* a longer estimate in time units (e.g., seconds) will be produced if the clock runs faster, because more clock pulses have been accumulated during the presentation of the time interval. In case of the *reproduction task,* a time interval is presented first and the participant is asked to reproduce it subsequently. In this task, the clock speed should have no effect on the mean estimates. The number of clock pulses accumulated during the presentation of the target time interval and the number of pulses accumulated during the production of the interval is equally affected if the clock runs fast (or slow). Thus, in a verbal estimation task, and only here, a time estimate that is higher than the actual duration of the presented time interval can be interpreted as an *overestimation* of time. In contrast, in the production task, a *shorter* produced time interval would represent an overestimation of time. For a reproduction task, the concept of general over- or underestimation of time does not apply.

In terms of the internal clock model, the subjective experience of a slow time flow, (Straus, 1947; Wyrick and Wyrick, 1977; Richter and Benzenhöfer, 1985; Münzel et al., 1988; Blewett, 1992; Bschor et al., 2004), which is different from explicit interval timing, could be attributed to a *faster* clock speed in depressed patients. For example, if both a depressed patient and a control subject have to wait for the experimenter for 5 min, then the number of clock pulses accumulated during these 5 min will be higher for the patient, which according to the model corresponds to a longer perceived time interval.

An alternative explanation for an effect of depression on time perception could be formulated in terms of attention (e.g., Sévigny et al., 2003). Drawing attention away from the timing task might result in the accumulator “missing” some clock pulses (cf. Zakay and Block, 1996). As a consequence, even if the clock speed is unaltered, distracting attention from the timing task should result in fewer pulses being accumulated during a given time interval and therefore the perceived time is shortened. This model is compatible with empirical findings regarding the effects of attention on time estimation (for a review see Brown, 2008). However, to explain the elevated verbal time estimates in depressed patients, it would be necessary to assume that they allocate more attention to the estimation task than do healthy controls.

Given the intricacies of measuring time perception, surprisingly few studies have used more than one method for obtaining estimates. Mezey and Cohen (1961) pioneered the comparative use of all three classical methods – estimation, production, and reproduction – with a narrow focus on 30-s-intervals. This work, however, has not been followed-up. Most recent studies have used a single task or method (e.g., Wyrick and Wyrick, 1977; Mahlberg et al., 2008). Some studies on temporal judgment were conducted in depressed patients only (e.g., Mezey and Cohen, 1961). These results are difficult to interpret, because in the three classical tasks systematic deviations of the time estimates from the veridical values are also observed for non-depressed participants (e.g., Wearden and Lejeune, 2008). Other studies included a control group, as for example healthy subjects or patients with clinical disorders other than depression (Grinker et al., 1973; Wyrick and Wyrick, 1977; Kitamura and Kumar, 1983; Tysk, 1984; Münzel et al., 1988; Bschor et al., 2004; Mahlberg et al., 2008). With this design, which was also used in our study, it is possible to detect effects of depression on time perception.

A recent review of existing studies on the effects of depression on time perception showed that the evidence is mixed at best (Msetfi et al., 2012). Some investigators found significant effects of depression in the direction predicted by a faster running internal clock, other studies found the opposite pattern, and several studies reported no significant effects. In the case of verbal estimation, some studies yielded results that indicate an overestimation of temporal intervals in depressive subjects compared to healthy control subjects (e.g., Wyrick and Wyrick, 1977; Kitamura and Kumar, 1983; Kornbrot et al., 2013). These results are in favor of the hypothesis of an increased-clock-speed in depression. However, other studies reported mixed results (Bschor et al., 2004; Biermann et al., 2011) or even contradictory evidence (underestimation in depressive patients relative to control subjects; Tysk, 1984). Likewise, data obtained by means of time production tasks are inconclusive. Beside statistically significant results that support the predictions of the clock model (underproduction in depressives; Mundt et al., 1998; Bschor et al., 2004), some studies did not report differences in the produced intervals between depressives and controls (Kitamura and Kumar, 1983; Tysk, 1984). Münzel et al. (1988) reported overproduction of time intervals in a depressive group compared to control subjects. Equally inconsistent results can be found in the context of time reproduction, which should not be affected by depression in the first place (e.g., Münzel et al., 1988; Mahlberg et al., 2008).

### SUBJECTIVE EXPERIENCE OF TIME

Apart from the laboratory tasks used to study time perception, an additional method is to directly ask for the participant’s subjective experience of the flow of the time (Wyrick and Wyrick, 1977; Richter and Benzenhöfer, 1985; Münzel et al., 1988; Blewett, 1992; Bschor et al., 2004). This estimate is different from the three classical interval timing tasks and might be sensitive to time experiences that fail to surface in time estimation or production tasks. Such a direct phenomenal impression of time perception has been obtained in the form of verbal statements, ratings, or visual analog scales (VASs). Three out of five studies found the subjective flow of time to be significantly slowed in depressive patients compared to healthy controls (Wyrick and Wyrick, 1977; Münzel et al., 1988; Bschor et al., 2004). However, the comparability of time experience measures and data from estimation and production tasks is questionable for several reasons. First, it has been proposed that depressed patients might be referring to a mood rather than to an experienced change in the flow of time when saying “time is passing by very slowly” (Bech, 1975; Münzel et al., 1988). Second, interval timing in estimation and production procedures usually applies to durations in the range of seconds or a few minutes, whereas the concepts of flow of time and time experience are rather related to less precisely defined and likely longer time intervals. We will provide an alternative account of the effects of depression on the subjective flow of time in the discussion section.

### RATIONALE OF OUR EXPERIMENT

Taken together, previous research shows only weak evidence for a difference between depressed patients and non-depressed subjects in the three classical time estimation tasks. In general, the different tasks have not been studied systematically across a wider range of time intervals. In particular, the subsecond range has not been studied sufficiently, and only few studies included more than one task. Due to the lack of empirical evidence from verbal estimation and production tasks at time intervals shorter than 1 s, it is difficult to judge whether the effect of depression on performance in the three classical time perception tasks – if present at all – might depend on the length of the interval. In the literature on time perception, it has frequently been suggested that short and long intervals are estimated via different mechanisms. For example, Fraisse (1984) proposed that a perceptual process is responsible for intervals shorter than 3 s (*perception of duration*), whereas a more cognitive process accomplishes the *estimation of durations* above 3 s. Grondin et al. (1999) found that the interval duration at which explicit counting becomes a useful strategy begins at 1.18 s. Ulbrich et al. (2007) confirmed this and proposed a temporal boundary in the range between 1 and 2 s. Additional evidence for multiple time estimation modes was provided by Grondin (2012) who found systematic changes in the Weber fraction between durations of 1 and 2 s. Thus, somewhere between 1 and 3 s, a qualitative difference in time perception has to be considered.

For these reasons, we presented time intervals of 0.5, 2, and 60 s in each of the three classical time perception tasks. Moreover, we obtained ratings of the subjective flow of time (e.g., Wyrick and Wyrick, 1977). To comprehensively address time perception, we systematically measured not only systematic deviations of the estimates from the veridical time duration (i.e., the constant error in terms of Fechner), but also considered the variable error as a measure of sensitivity. The variable error was defined as the standard deviation of the verbal estimates or produced duration across the trials presenting a given target interval. This measure should be closely related to results from duration discrimination tasks (Treisman, 1963), where typically two almost identical temporal intervals are presented successively, and the subject is asked to judge which of the two intervals was longer (or shorter). The sensitivity in this task is typically expressed in terms of the difference limen denoting the difference in duration between two stimuli that results in for example 75% correct responses. The evidence concerning effects of depression on duration discrimination is inconclusive. Depending on the study and on the duration of the intervals, compared to non-depressed persons smaller, larger, or equal difference limens were reported for depressed persons (Rammsayer, 1990; Sévigny et al., 2003; Msetfi et al., 2012).

Another innovation of our study was the inclusion of *TTC judgments* (cf. Hecht and Savelsbergh, 2004), which have thus far not been used as a time perception measure in depressed patients. The added TTC estimation task allows for a comparison of potential effects of depression on time estimation vs. motor action. Motor action involves an ability of temporal processing that may draw on different timing mechanisms than do explicit judgments. Vierordt’s (1868) law describes the observation that productions of short durations are on average longer than the veridical value, but the productions of longer durations tend to be shorter than the presented time interval. This pattern is observed in classical timing tasks (Wearden and Lejeune, 2008) as well as in PM tasks, where the cross-over point is at 1 or 2 s, above which TTC is being underestimated (Schiff and Detwiler, 1979; Manser and Hancock, 1996; Oberfeld and Hecht, 2008). Additionally, the variability of the estimates relative to the mean of the estimates or to the nominal value (i.e., Weber fractions) was frequently reported to differ between short, medium, and long interval durations, both for the classical timing tasks (cf. Wearden and Lejeune, 2008; Grondin, 2012), duration discrimination (e.g., Oberfeld, 2014), and for TTC estimates in a PM task (e.g., Oberfeld and Hecht, 2008). For these reasons, in all of the four tasks, we obtained sufficiently high numbers of trials to be able to analyze not only the mean time estimates but also their variability because the latter measures bear importance for models of time perception (Wearden and Lejeune, 2008).

If the assumption of altered time perception in depressive patients in terms of a faster running internal clock is true, we expect the subjects in the depressive group to underproduce and overestimate time intervals in the time production task and the verbal time estimation task, respectively, compared to the control subjects. In the time reproduction task, however, no differences are expected. TTC estimates should be shorter in the depressive group due to the discussed similarity to a time production task.

## MATERIALS AND METHODS

### PARTICIPANTS

Forty-eight volunteers (24 patients and 24 controls) participated in the study. The data of two controls and two patients were excluded from the final analysis due to an unknown history of psychological illness (*N* = 1), an extremely high depression score in a subject from the control group (*N* = 1), incomplete data (*N* = 1), or extreme slowness of performance (*N* = 1). Patients (5 males and 17 females) ranged in age between 19 and 53 years (*M*= 35.23 years, *SD* = 10.92) and controls (11 males and 11 females) ranged between 19 and 37 years (*M*= 25.03 years, *SD* = 4.58). Seven patients reported to be on medication. All subjects gave informed written consent according to the Declaration of Helsinki. The local ethics committee had approved the study. All subjects had normal or corrected-to-normal vision. Depression patients (primary diagnosis) were recruited by a clinical psychologist prior to their treatment at the psychotherapeutic outpatient unit at the University of Mainz. They were screened using the IDCL-Internationale Diagnosen Checklisten (Hiller et al., 1997). Patients met the criteria for major depression based on DSM-IV. Patients with a past or present history of organically induced psychological illness, substance abuse, schizophrenia, personality disorders, acute suicide ideation, and post-traumatic stress disorder were excluded from the study. Control subjects were students from the University of Mainz. They reported to have no significant past or present history of psychological illness.

All subjects filled in the beck depression inventory (BDI)-German version (Hautzinger et al., 1994). This inventory consists of 21 items and uses four-point rating scales (0–3). The higher the score on each item, the higher the probability of depression. Participants rated how well the items (e.g., “I feel sad most of the time”) described how they had felt for a week before the experiment. A total score between 0 and 11 indicates no significant depression, values higher than 11 indicate mild depression, and values higher than 17 indicate clinically relevant depression (Hautzinger et al., 1994). According to the test’s manual, Cronbach’s α of the scale was 0.87 in depressed patients, and 0.74 in healthy controls. In our sample of depressive patients, Cronbach’s α was 0.81, and in healthy controls it was also 0.81. The total BDI scores were significantly higher in the patient group (*M* = 21.45, *SD* = 7.28) compared to the control group (*M* = 4.86, *SD* = 4.41),* t*(42) = 9.141, *p* < 0.001, Cohen’s (1988) *d* = 2.8.

### DESIGN

The experiment comprised four tasks: verbal time estimation, time production, time reproduction, and TTC judgment. In the three classical time perception tasks, time intervals were estimated or produced prospectively (i.e., subjects were informed about the temporal task in advance). In a repeated measures design, the patient group and the control group received all of the four tasks. The verbal time estimation task was always administered in the first block. The reason for this task order was that in the time production task, time intervals of 0.5, 2, and 60 s were specified numerically on the screen. We were concerned that when presenting the production task before the verbal estimation task, subjects might use these numerical time values as anchors (cf. Tversky and Kahneman, 1974) when producing verbal time estimates. Presenting the verbal estimation task in the first block avoided this problem. For the three remaining tasks, subjects were randomly assigned to the six possible presentation orders. In addition, all subjects rated the subjective flow of time on a VAS, and provided a retrospective time judgment of the time spent in the lab.

### APPARATUS

The four temporal tasks were programmed using Vizard VR Tool 3.18 (2011). The stimuli were presented on a DELL TFT display (width of 31 cm, and height of 23 cm) with a resolution of 1024 × 768 pixels and a colour depth of 32 bits. The display was positioned inside a box (width of 98 cm, height of 98 cm, and depth of 120 cm). The subject’s head was steadied by a chin rest to maintain a viewing distance of 50 cm from the screen.

### PROCEDURE

All subjects were tested after 12 p.m. (Kitamura and Kumar, 1983; Tysk, 1984; Richter and Benzenhöfer, 1985; Münzel et al., 1988; Kuhs et al., 1989; Blewett, 1992; Bschor et al., 2004) to avoid effects of diurnal variation. As soon as the subjects had arrived outside the experiment room, they were asked to remove their watches (without looking at the time) and to enter the room. This entry time was noted by the experimenter. After collecting informed consent, the subjective experience of the flow of time on the day of experimentation was recorded. The subject was asked to indicate experienced flow of time on a VAS, consisting of a horizontal 100 mm line (Bschor et al., 2004) that represented time experience from “as slowly as possible” (0 mm) to “as quickly as possible” (100 mm). Then, subjects filled in a socio-demographic and a clinical data sheet. Then they received written instructions, time for addressing the experimenter in case of questions, and six practice trials, separately before each session. The subject was seated comfortably in front of the display screen.

#### Verbal time estimation task

At trial start, the screen was black. After pressing a button, the complete screen turned white for 0.25 s (flash 1), then it turned black, and white again for another 0.25 s (flash 2). The inter-onset intervals (IOIs) between the two flashes were 500 ms, 2, or 60 s. The subject then entered his or her estimate of the time span between the two flashes in seconds with two digits after the decimal point (e.g., “0.25 s”), using a computer keyboard. We did not instruct the subjects to avoid counting. Each subject received the short intervals (500 ms, and 2 s) on 15 trials each, and the long interval (60 s) on seven trials, in random order, resulting in a total of 37 trials.

#### Time production task

On each trial, an instruction appeared on the screen asking the candidate to produce a time interval of 0.50, 2, or 60 s. The subject pressed the spacebar to indicate the beginning of the interval, and pressed the spacebar again to indicate its end. The time intervals and trial numbers were the same as in the verbal estimation task.

#### Time reproduction task

The stimulus for the reproduction task was the same as in the verbal estimation task (empty visual time interval). Once the interval had been presented, the subject was asked to produce it from memory by pressing the spacebar two times, just as in the time production task. The time intervals and trial numbers were the same as in the production and estimation task.

#### Prediction-motion (time-to-contact) task

In the PM task, a disk was presented on the display, moving horizontally from the left to the right side of the screen (i.e., motion on the frontoparallel plane) at constant speed toward a finishing line located at the right edge of the display. The disk was black, shown against a light gray background. Its diameter was 1.0 cm (visual angle of 1.15°), its velocity 3 or 9 cm/s. After 800 ms, the disk disappeared from the screen. Subjects were asked to press a response key at the instant when the disk would have collided with a vertically oriented black arrival line (Schiff and Detwiler, 1979). The TTC was defined as the time interval between the disappearance of the disk from the screen and the instant when the disk would have reached the arrival line if it had continued its constant-speed trajectory. Three TTCs were presented (0.5, 1.25, and 2.0 s). For each velocity, the starting position was selected to produce the designated TTC. Each velocity × TTC combination was presented 20 times in random order. The difference in time between the disappearance of the disk from the screen and the key press was taken as the TTC estimate.

#### Retrospective time judgment

Once the subject had completed the four time judgment tasks, the BDI was administered. Finally, the subjects reported a retrospective judgment (in minutes) of the time elapsed since their entry into the room, and the experimenter noted the actually elapsed time.

## RESULTS

For each subject × task × time interval combination, observations more than three times the interquartile range below the first quartile or above the third quartile were classified as outliers (Lovie, 1986), and were excluded from the analysis. In only 5 of the 396 combinations more than one outlier was excluded. In total, 1.5% of the trials were excluded. For each subject and condition, we computed the mean relative error, defined as the difference between the estimated time interval and the veridical time interval, divided by the veridical time interval (for a discussion of different error measures see Kornbrot et al., 2013). A positive value of the mean relative error indicates that the subjective estimate was greater than the veridical time interval, a negative value stands for a smaller estimate, and zero indicates that the estimate made by the subject was accurate.

### CLASSICAL TIMING TASKS

#### Relative error

**FIGURE 1:**
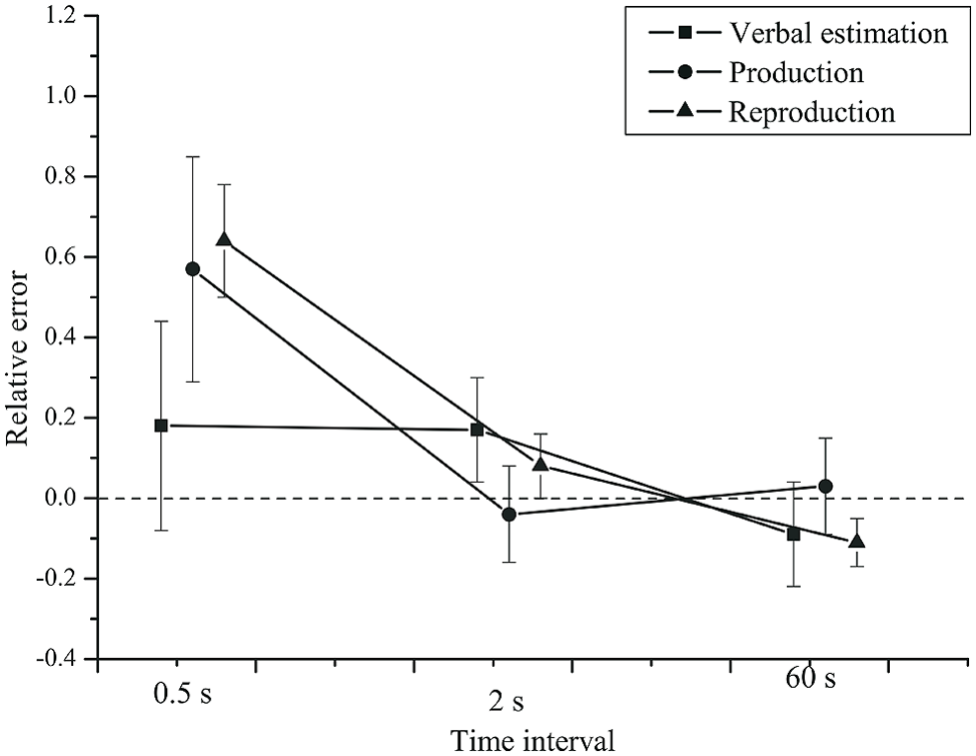
**Mean relative error as a function of time interval and task.** Squares: verbal estimation. Circles: production. Triangles: reproduction. Error bars show 95% confidence intervals.

First, we compared the three classical methods. As visible from **Figure 1**, the mean estimates were higher than the veridical values at the 0.5 s time interval. For longer time intervals the relative error was zero or the estimates were slightly lower than the veridical values. We conducted a repeated-measures analysis of variance (rmANOVA) on the relative error, using a univariate approach with Huynh-Feldt correction for the degrees of freedom (Huynh and Feldt, 1976). The correction factor 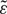 is reported and partial η^2^ is used as a measure of effect size. The within-subjects factors were task (verbal estimate, time production, time reproduction) and time interval (0.5, 2, 60 s), and the between-subjects factor was group (depressive patients and healthy controls).

Time interval had a significant effect on the relative error, *F*(2,84) = 37.85, *p* < 0.001, $\eta_{p}^{2}$ = 0.47, 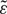 = 0.63. The relative error was higher for the 0.5 s interval (*M* = 0.46, *SD* = 0.47) than for the 2 s (*M* = 0.07, *SD* = 0.19) and the 60 s (*M* = -0.06, *SD* = 0.15) intervals.

The task × time interval interaction was significant, *F*(4,168) = 7.73, *p* = 0.002, $\eta_{p}^{2}$ = 0.16, 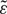 = 0.41. At the 0.5 s interval, the relative error was greater than for 2 and 60 s intervals in the production and the reproduction task, but not for the verbal estimation task. Potential origins of this duration-dependent effect of task are discussed below.

**FIGURE 2:**
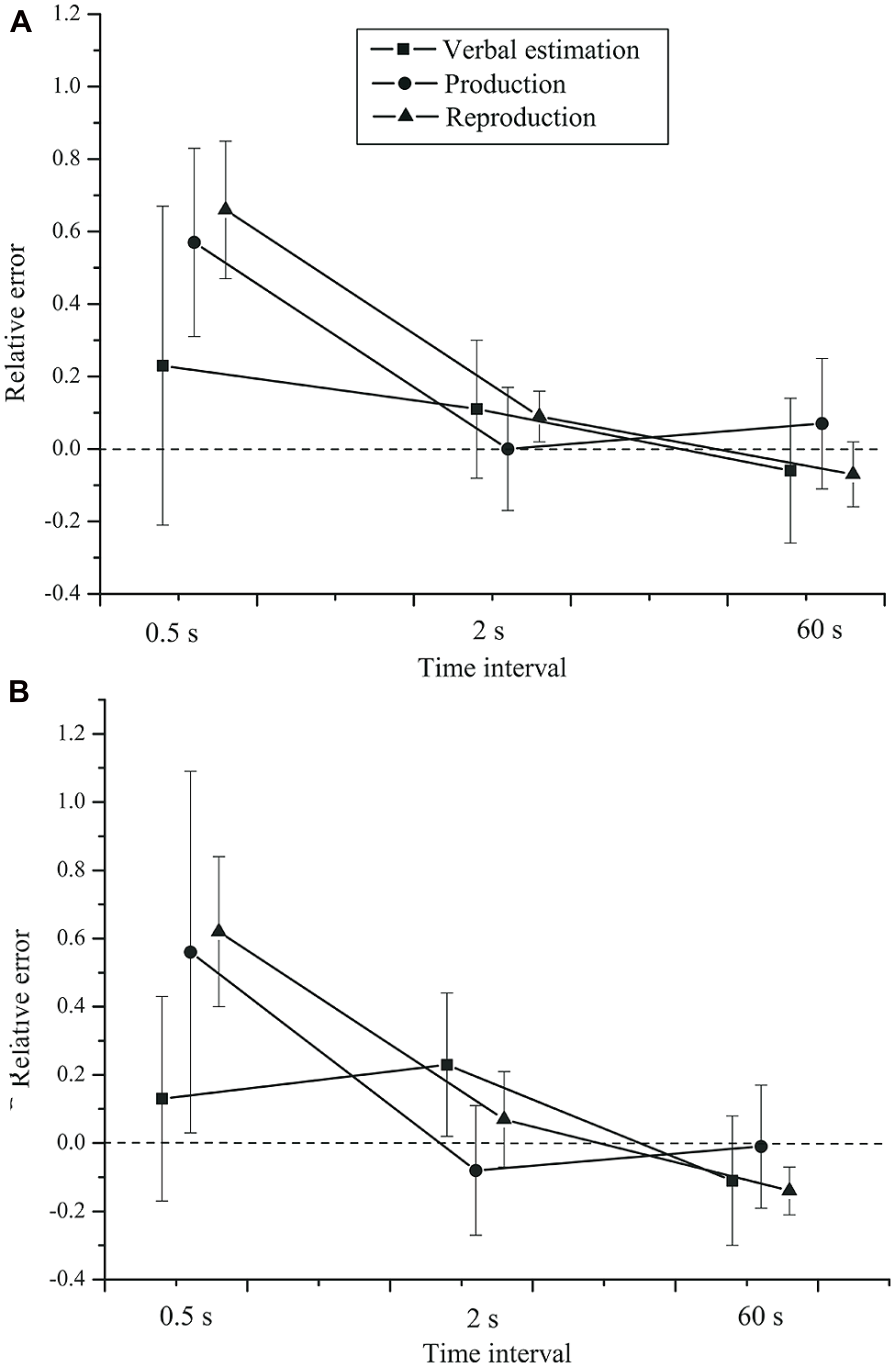
**Mean relative error as a function of time interval, task, and group.** **(A)** Control group. **(B)** Depressive patients. Error bars: 95% CIs.

The effect of task on the relative error, *F*(2,84) = 0.983, *p* = 0.379, $\eta_{p}^{2}$ = 0.023, did not reach significance. No significant group × time interval interaction, *F*(2,84) = 0.19, *p* = 0.721, $\eta_{p}^{2}$ = 0.005, or group × task interaction, *F*(2,84) = 0.04, *p* = 0.886, $\eta_{p}^{2}$ = 0.001 (see **Figure 2**) were found. Neither was there a task × time interval × group interaction, *F*(4,168) = 0.346, *p* = 0.664, $\eta_{p}^{2}$ = 0.008. Finally, the main effect of group was not significant, *F*(1,42) = 0.445, *p* = 0.508, $\eta_{p}^{2}$ = 0.010.

Thus, all tasks showed the same pattern, but for short intervals the average estimates were more accurate in the verbal estimation than in the time production and reproduction tasks. At short intervals, the relative error was smaller in the verbal estimation task. For longer intervals, the three classical timing tasks yielded very comparable results. Most importantly, we found no effects of depression on the relative error.

#### Variable error

The variable error was defined as the standard deviation of the individual responses for a given task and time interval. We analyzed the relative standard deviation (i.e., standard deviation divided by time interval), which can be described as a Weber fraction. A high value of the Weber fraction indicates that the responses of a subject varied strongly across the 7 to 15 trials collected per combination of task and time interval. For three classical tasks, a rmANOVA was conducted. The within-subjects factors were task and time interval, and the between-subjects factor was group (depressive patients and controls).

**FIGURE 3:**
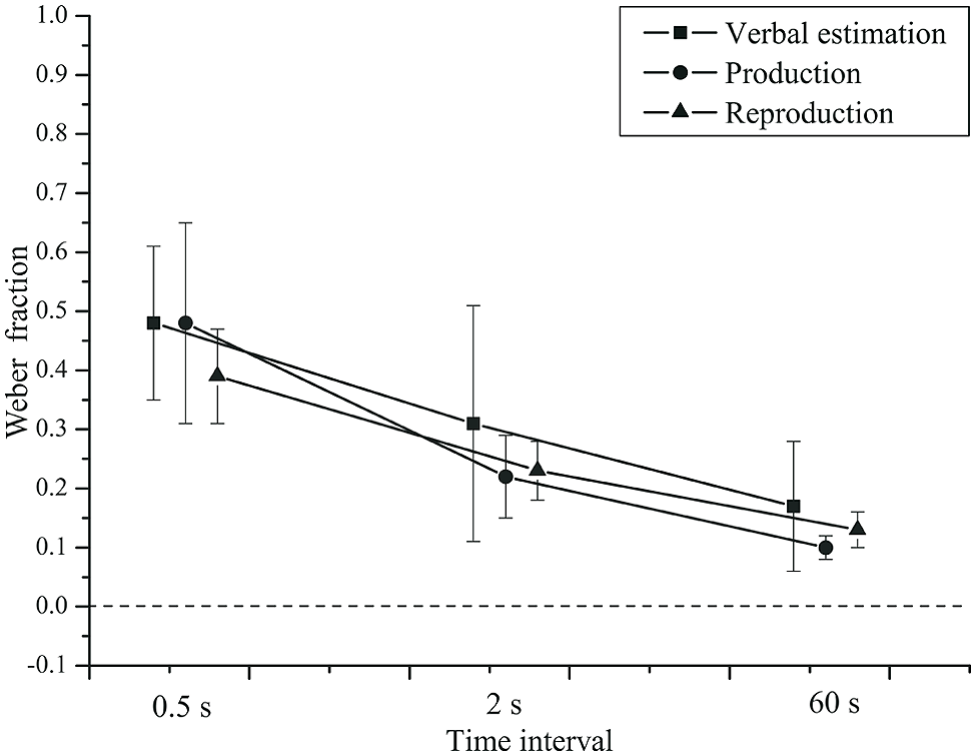
**Mean Weber fractions as a function of time interval and task.** Error bars: 95% CIs.

**FIGURE 4:**
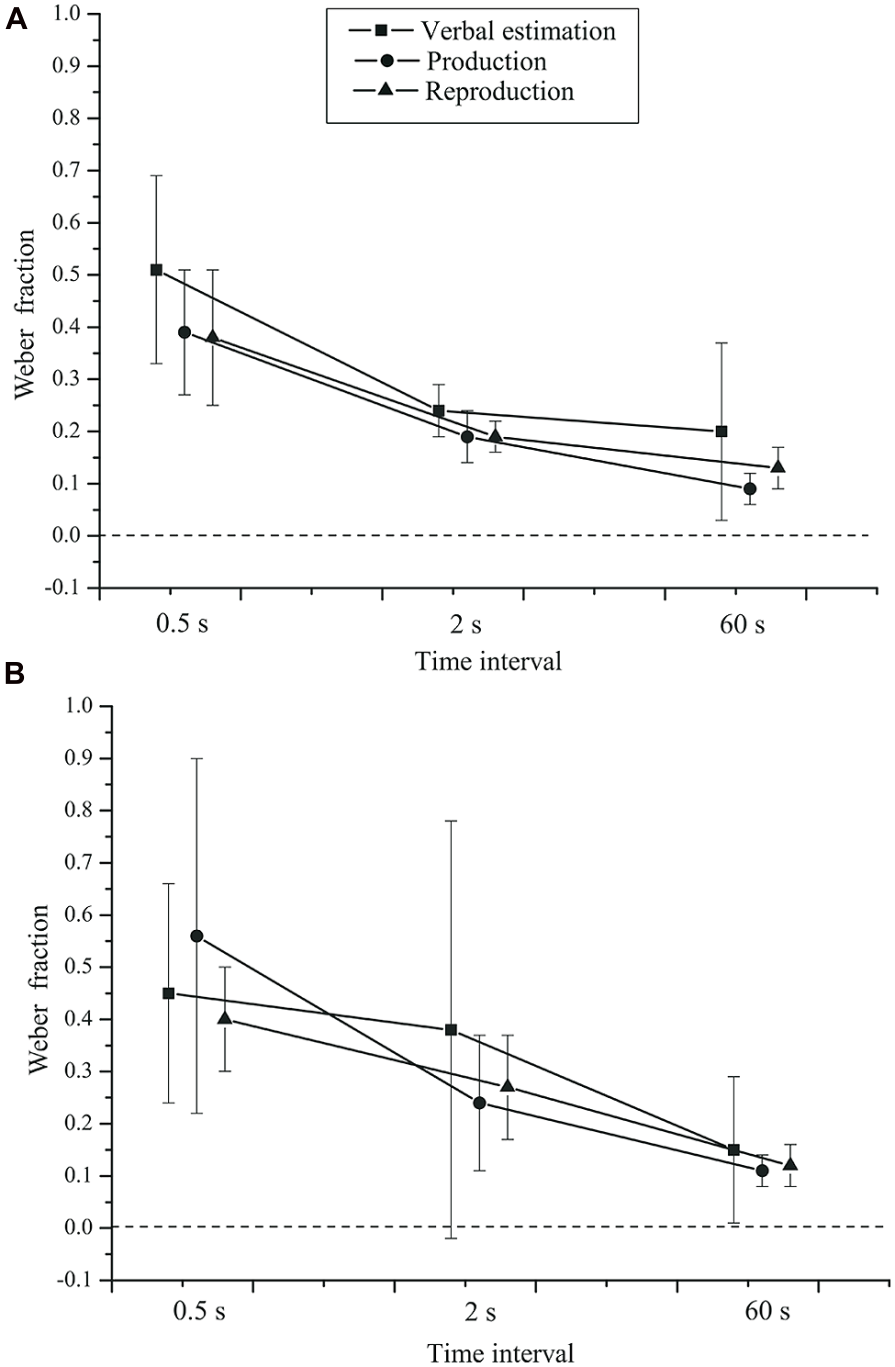
**Mean Weber fractions as a function of time interval, task, and group.** **(A)** Control group. **(B)** Depressive patients. Error bars: 95% CIs.

Time interval had a significant effect (see **Figure 3**) on the Weber fraction, *F*(2,84) = 34.429, *p* < 0.001, $\eta_{p}^{2}$ = 0.450, 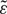 = 0.963. The Weber fraction was highest for the 0.5 s interval (*M* = 0.45, *SD* = 0.32), followed by the 2 s (*M* = 0.25, *SD* = 0.28) and the 60 s (*M* = 0.13, *SD* = 0.13) intervals. The task × group interaction, *F*(2,84) = 0.243, *p* = 0.680, $\eta_{p}^{2}$ = 0.006, and the time interval × group interaction, *F*(2,84) = 0.877, *p* = 0.416, $\eta_{p}^{2}$ = 0.020, were not significant (see **Figure 4**).

There was no effect of task, *F*(2,84) = 0.988, *p* = 0.344, $\eta_{p}^{2}$ = 0.023, 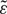 = 0.633. Neither was the task × time interval × group interaction significant, *F*(4,168) = 1.148, *p* = 0.330, $\eta_{p}^{2}$ = 0.027. The main effect of group was also not significant, *F*(1,42) = 0.391, *p* = 0.535, $\eta_{p}^{2}$ = 0.009.

### TIME-TO-CONTACT JUDGMENT

#### Relative error

**FIGURE 5:**
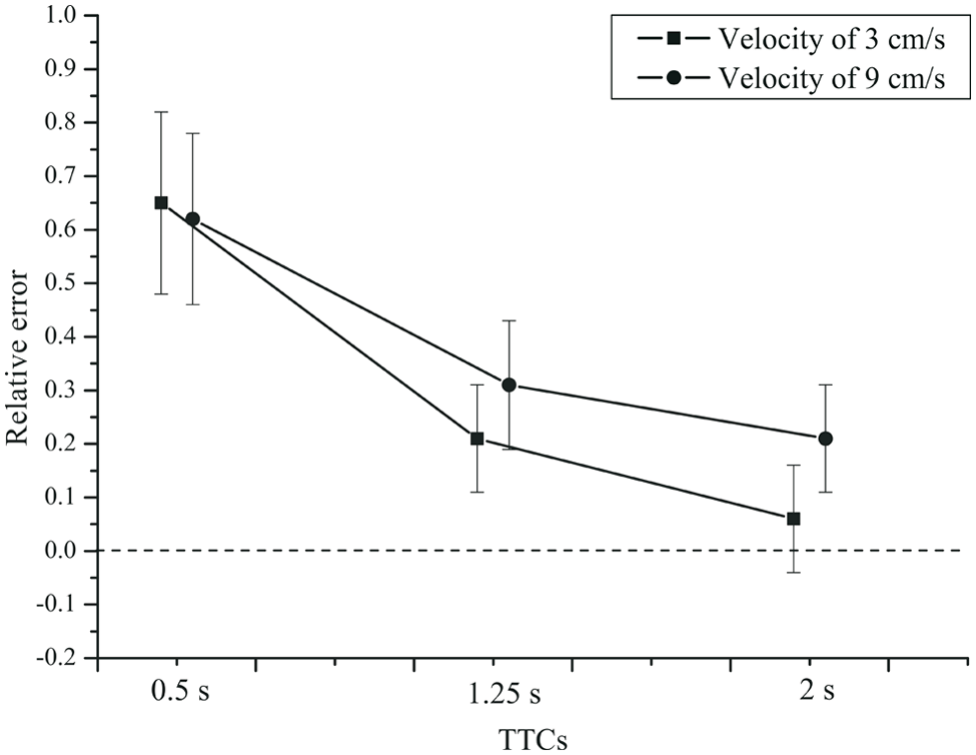
**Mean relative error of the TTC estimates, as a function of TTC and velocity.** Error bars: 95% CIs.

**FIGURE 6:**
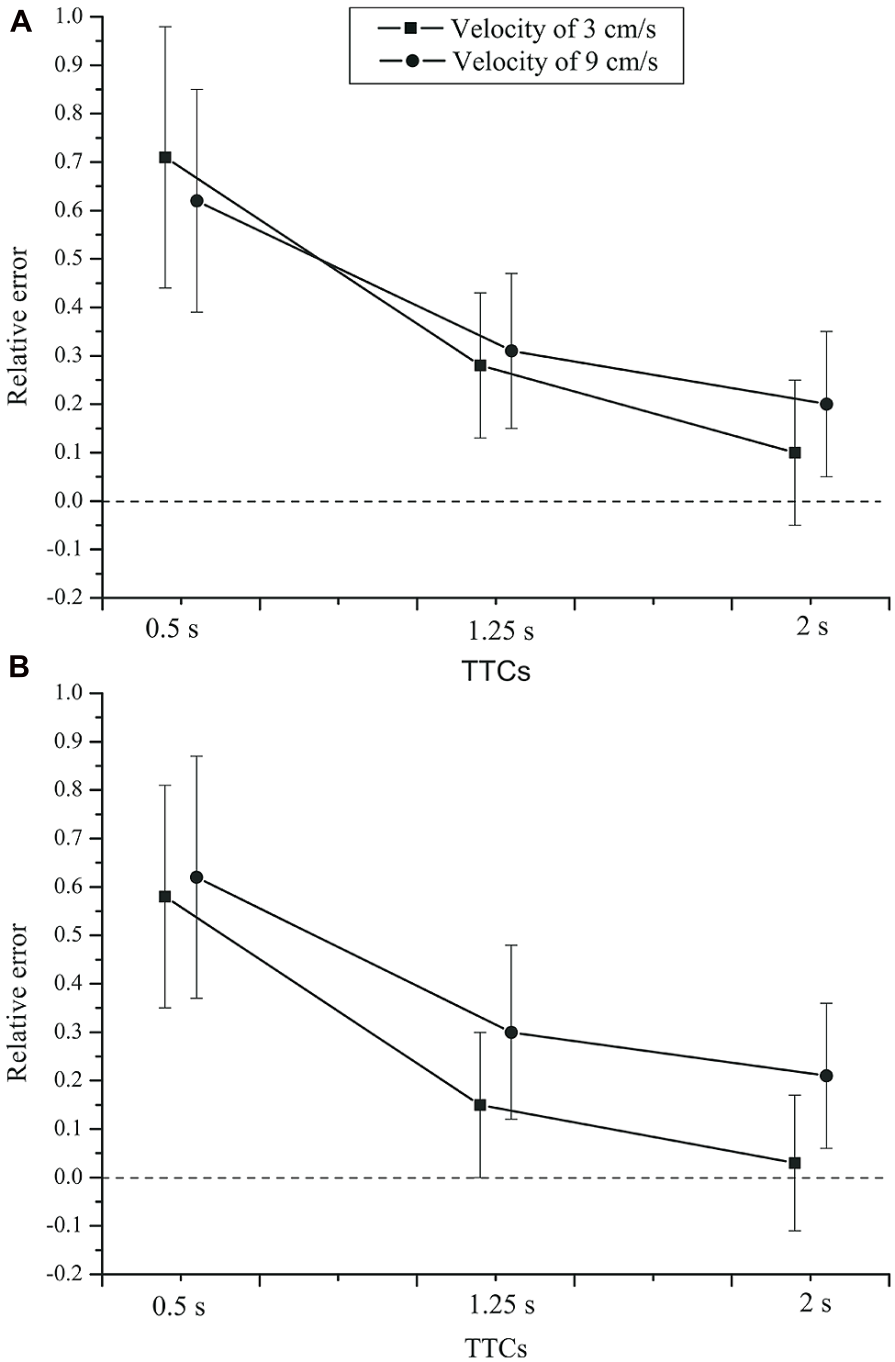
**Mean relative error of the TTC estimates as a function of TTC, velocity, and group.** **(A)** Control group. **(B)** Depressive patients. Error bars: 95% CIs.

For the TTC estimation (PM) task, we used the same criterion for outlier exclusion as for the timing tasks. In total, only 0.2% of the trials were excluded as outliers. An rmANOVA with the within-subjects factors velocity and TTC, and the between-subjects factor group (patients and controls), showed a significant effect of TTC on the relative error, *F*(2,84) = 76.122, *p* < 0.001, $\eta_{p}^{2}$ = 0.644, 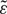 = 0.551. The mean relative errors (see **Figure 5**) show that TTC estimates were generally above the veridical value, especially at a TTC of 0.5 s. Velocity had no significant effect on the relative error of the TTC estimates, *F*(1,42) = 3.790, *p* = 0.058, $\eta_{p}^{2}$ = 0.083. For the slower velocity (3 cm/s), the TTC tended to be judged shorter than for the faster velocity (9 cm/s). A significant velocity × TTC interaction was found, *F*(2,84) = 7.831, *p* = 0.003, $\eta_{p}^{2}$ = 0.157, 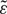 = 0.692. Only for the two longer TTCs, the relative error was lower for the fast compared to the slow velocity. The velocity × group interaction was not significant, *F*(1,42) = 2.553, *p* = 0.118, $\eta_{p}^{2}$ = 0.057. There was no TTC × group interaction,* F*(2,84) = 0.095, *p*= 0.784, $\eta_{p}^{2}$= 0.002 (see **Figure 6**). Neither was there a velocity × TTC × group interaction, *F*(2,84) = 0.182, *p* = 0.751, $\eta_{p}^{2}$ = 0.004. The main effect of group was not significant, *F*(1,42) = 0.214, *p* = 0.646, $\eta_{p}^{2}$ = 0.005.

#### Variable error

**FIGURE 7:**
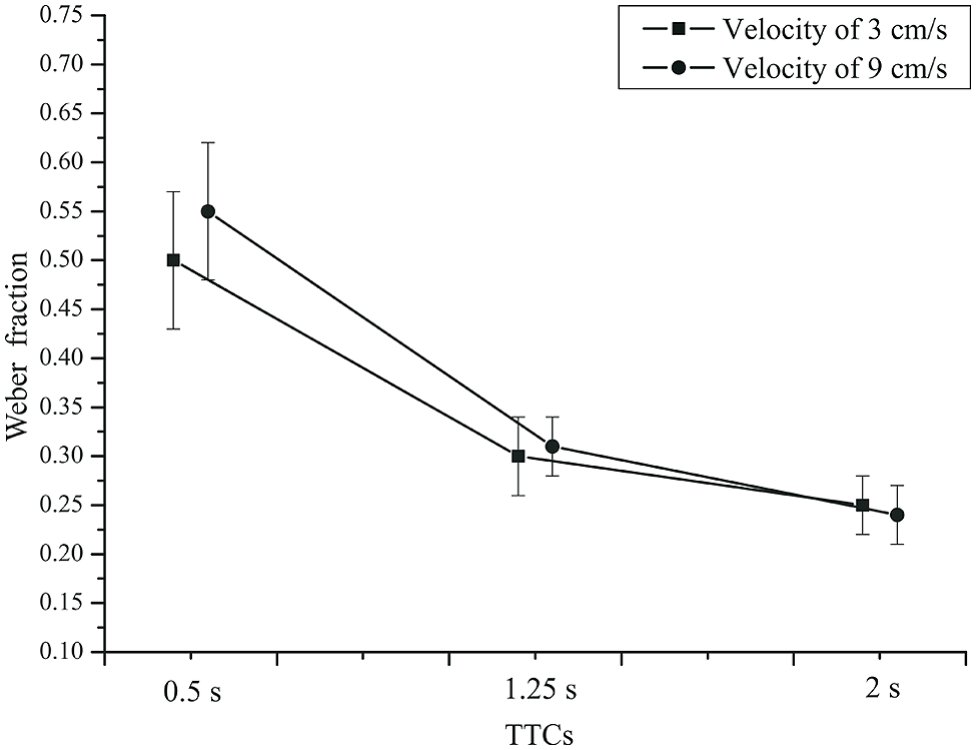
**Mean Weber fractions as a function of TTC and velocity.** Squares: 3 cm/s. Circles: 9 cm/s. Error bars: 95% CIs.

**FIGURE 8:**
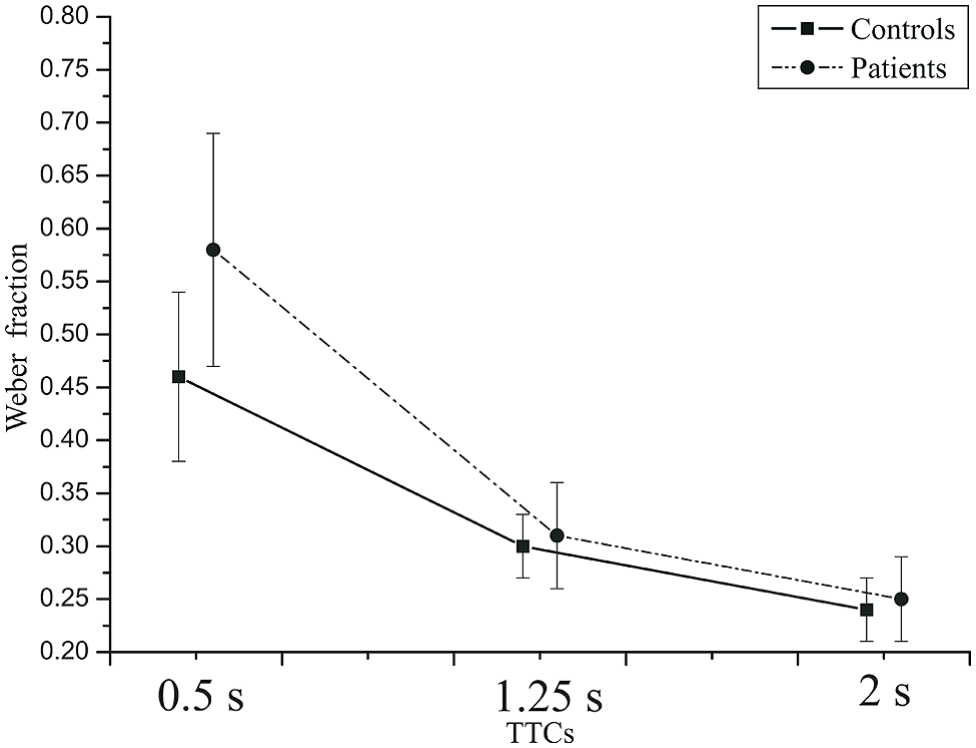
**Mean Weber fractions as a function of TTC in controls (squares) and patients (circles).** Error bars: 95% CIs.

**FIGURE 9:**
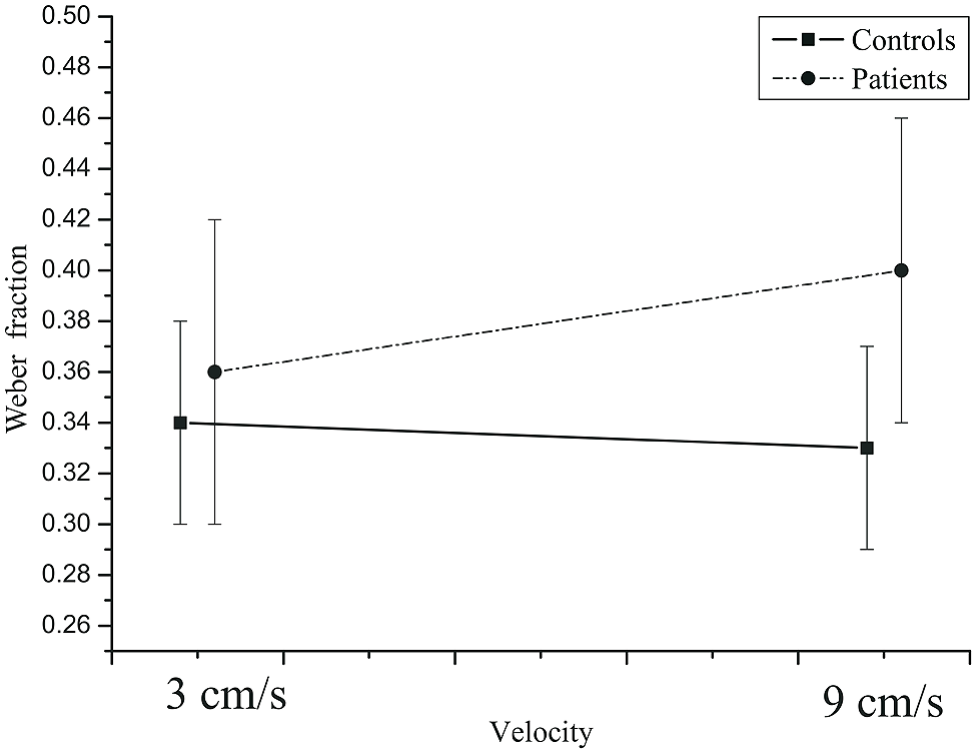
**Mean Weber fractions as a function of velocity in controls and patients.** Error bars: 95% CIs.

We also analyzed the variability of TTC judgments by computing Weber fractions (standard deviation divided by TTC). An rmANOVA with the within-subjects factors velocity and TTC, and the between-subjects factor group (patients and controls) showed a significant effect of TTC on the Weber fractions, *F*(2,84) = 83.677, *p* < 0.001, $\eta_{p}^{2}$ = 0.666, 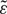 = 0.581. The Weber fraction was larger for short compared to long TTCs (refer to **Figure 7**). This is compatible with earlier reports (DeLucia and Liddell, 1998; Oberfeld and Hecht, 2008). The velocity × TTC interaction was significant, *F*(2,84) = 4.669, *p* = 0.018, $\eta_{p}^{2}$ = 0.100, 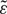 = 0.809. For the two shorter TTCs, the Weber fraction was larger for the faster than for the slower velocity. Velocity did not have a significant main effect, *F*(1,42) = 0.895, *p* = 0.349, $\eta_{p}^{2}$ = 0.021. The effect of group was not significant, *F*(1,42) = 2.02, *p* = 0.16, $\eta_{p}^{2}$ = 0.046. Thus, depression patients did not produce more variable TTC estimates than did healthy controls. The TTC × group interaction just failed to reach significance, *F*(2,84) = 3.61, *p* = 0.058, $\eta_{p}^{2}$ = 0.079, 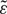 = 0.581. At the 0.5 s TTC, the Weber fractions were higher in patients than in the controls, whereas at the two longer TTCs, there was not much difference between the two groups (**Figure 8**). The velocity × group interaction was not significant, *F*(2,84) = 3.28, *p* = 0.078, $\eta_{p}^{2}$ = 0.072, 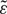 = 1.0. At the slow velocity, the Weber fractions were almost identical in patients and controls. However, at the fast velocity, the Weber fraction was higher in patients than in controls (**Figure 9**).

Thus, TTC estimation performance varied with the duration of the time interval until collision. Relative errors were largest for short TTCs and diminished with longer TTCs. Note that this pattern is more similar to the production and reproduction tasks than to the temporal estimation task. Overestimation of TTC was found throughout. Weber fractions were higher at short compared to long TTCs. On average, depressive patients and controls did not produce different TTC estimates, but in the two groups the Weber fractions depended on TTC and velocity in a slightly different manner.

### SUBJECTIVE EXPERIENCE OF THE FLOW OF TIME AND RETROSPECTIVE TIME ESTIMATION

The subjective experience of the flow of time indicated by the VAS (100 mm) was not significantly faster for controls (*M* = 62.32 mm, *SD* = 18.91 mm) than for patients (*M* = 61.32 mm, *SD* = 22.27 mm), *t*(42) = 0.161, *p* = 0.87.

The time that actually elapsed between entering the laboratory and the administration of the retrospective time judgment ranged between 65 and 93 min (*M* = 78.7 min, *SD* = 6.9 min). The estimate of this time was analyzed in terms of the relative error as for the other tasks. The depressive patients showed a slightly stronger tendency toward underestimation (mean relative error = -0.24, *SD* = 0.16) than the controls (*M* = –0.19, *SD* = 0.14), but this difference was not significant, *t*(42) = 1.17, *p* = 0.66.

### CORRELATIONS BETWEEN BDI SCORES AND MEASURES OF TIME PERCEPTION

As shown in **Table 1**, the relative errors and the Weber fractions on the three classical timing tasks, the TTC estimation task, and the retrospective time judgment were not significantly correlated with the depression severity scores obtained on the BDI. The VAS rating of the subjective flow of time was also uncorrelated to the BDI score. All *p* values were higher than 0.1, and all *r*-values were below 0.25.

**Table 1:** Correlations between the relative errors or Weber fractions and the total BDI score, with two-tailed *p*-values.

	Task					
	Production	Reproduction	Verbal estimation	TTC estimation	Retrospective time judgment	Flow of time
Relative error	*r* = -0.18 *p* = 0.243	*r* = -0.15 *p* = 0.345	*r* = 0.05 *p* = 0.771	*r* = -0.08 *p* = 0.593	*r* = -0.11 *p* = 0.480	[VAS rating] *r* = 0.14 *p* = 0.366
Weber fraction	*r* = 0.09 *p* = 0.560	*r* = 0.17 *p* = 0.276	*r* = 0.20 *p* = 0.205	*r* = 0.23 *p* = 0.128	–	–

*The column Flow of time shows the correlation between the rating on the VAS and the BDI score. *N* = 44.*

## DISCUSSION

We have investigated whether explicit time perception and time–to-contact estimation differ systematically between depressive patients and healthy control subjects. According to the internal clock model, the clock speed in depressed patients might be faster than in controls, systematically affecting performance in timing tasks. We have employed the three classical tasks of verbally estimating, non-verbally producing, and reproducing a specified time interval, as well as a TTC estimation task. We also assessed the participants’ subjective flow of time on the day of the experimentation. The time estimates depended on the task and the interval duration, but not on the depression status (patients vs. controls). Patients’ time perception, TTC estimation, and time experience did not differ systematically from that of healthy controls. The only exception was a tendency toward a slightly different effect of motion parameters on the Weber fraction in the TTC estimation task.

The relative errors obtained in the three classical tasks revealed no differences between depressed patients and controls. This result is in line with approximately half of the earlier reports (cf. Msetfi et al., 2012). The relative error was affected by the duration of the time interval to be judged. Consistently, the duration of very short intervals below 1 s tended to be overestimated. Here, the task was critical: Verbal estimation was less prone to error than tasks including a motor component. An explanation for this effect could be a memory rectification in the purely verbal task (as proposed by Sévigny et al., 2003) or a detrimental effect of motor activation of the fingers that might have caused a delay and thereby higher estimates in the production and reproduction tasks, which was absent in the verbal estimation task. The motor delay should be independent of interval duration (e.g., Wing, 1980) and thus produce a constant effect that is relatively stronger at shorter durations. It should be noted that the amount of additional variance induced by the motor component can be assumed to be independent of the interval duration (e.g., Wing, 1980). For this reason, the relative effect of motor variability on the variable error in the production and reproduction tasks should be strongest for short durations, as observed in the experiment (see **Figure 3**). However, the variable error showed the same dependence on interval duration for the verbal estimates, where motor variance should play no role. Thus, the motor variance does not appear to be the critical factor for the variable error.

How do the observed relative errors and Weber fractions compare to previous studies? For a production task, Wearden and Lejeune (2008) reported relative errors of approximately 0.4 when a 0.5-s-interval was asked for, whereas 1-s-intervals were produced close to perfection. For verbal time estimation, they observed relative errors around 0.0 at both durations. This pattern is very similar to our findings (see **Figure 1**). The observed decrease in the Weber fraction with interval duration (see **Figure 3**) is compatible with data by Treisman (1963), who presented interval durations of 0.5 and 2 s in a production and in a reproduction task. However, the Weber fractions in the present study were two to three times higher than in Treisman (1963). Grondin (2012) also reported a smaller Weber fraction (0.08) for a 1.9 s interval in a reproduction task.

For the implicit timing task using TTC judgments, the picture was similar. The relative error did not differ between depressed patients and controls. Relative TTC estimation errors did vary as a function of the actual time remaining until collision. The TTC overestimation was stronger for the shorter TTC (0.5 s) than for longer TTCs, which is consistent with previous results in PM tasks (Schiff and Detwiler, 1979; Manser and Hancock, 1996; Oberfeld and Hecht, 2008). Unlike the relative error measures, the variable error did differ slightly between patients and healthy controls. We observed a higher Weber fraction in depressed patients than in controls at the faster velocity (9 cm/s) and at the shortest TTC (0.5 s). At this point, we do not know whether this slightly elevated variability in patients is a direct or indirect consequence of depression. It could be mediated by a lack of attention induced by depression. Across conditions, the Weber fractions obtained in the TTC tasks were similar to earlier data (DeLucia and Liddell, 1998; Oberfeld and Hecht, 2008).

Taken together, our data provide no evidence indicating that depression leads to a systematic distortion of time perception or of time experience. Only in the perception-action task of judging TTC, a noticeable trend was found. And this trend was limited to variability of the estimates. With our sample size of 22 subjects per group, we had a 0.74 power (1 - β) to detect large-sized effects (*d* = 0.8) of depression for each combination of task and measure (relative error, Weber fraction), but only a 0.36 power to detect medium-sized effects (*d* = 0.5). The overall power of our study was higher, however, because we studied six different tasks, four different time intervals, and two different response measures (relative error and Weber Fraction). Our data thus suggest that effects of depression on time perception and TTC estimation are less than medium-sized. It might of course be possible to detect small effects of depression on time perception with much larger samples.

Our study tested patients with clinically relevant depressive symptoms, as evident in the high BDI scores. This is an advantage over studies that compared subjects with high and low BDI scores in subclinical samples. However, there are some methodological limitations, which might have contributed to the lack of evidence for differences between depressed and non-depressed participants in our data. First, the mean age differed between control group and patient group, and the gender composition of the two groups was different. A meta-analytic review on age effects on timing by Block et al. (1998) provides evidence for age-related differences in interval timing. However, these differences have not been reported to occur in midlife, but rather when comparing young adults to much older adults, for example at age 60 or 70, who already show a decline in executive functions and attentional processes (Aartsen et al., 2002; Balci et al., 2009). Because in our sample the mean age was 25 and 35 years for the control and patient groups, respectively, and the oldest participant was 52 years old, the age differences are unlikely a confounding variable. Even if age-related effects had interfered with the effects of depression on time perception in our sample, they would have caused a bias to *overestimate* the effects of depression. In particular, the tendency of older adults to overestimate and underproduce temporal intervals (Block et al., 1998) would have been a confound for the predicted effects of depression, but cannot explain our results indicating no effect of depression.

The somewhat higher proportion of female participants in the patient group (77%) compared to the control group (50%) might have had an effect on the results, however. There is evidence for a small gender effect on timing performance, with female participants giving more attention to time in prospective estimation tasks and displaying better episodic memory in retrospective paradigms [see Block et al. (2000) for a meta-analytic review]. Another methodological constraint of our study was that our student sample was rather homogeneous, whereas the depressed participants were drawn from a broader community. Thus, effects of potential differences between the groups regarding educational level, IQ, familiarity with the laboratory setting, and motivation cannot be ruled out. However, to our knowledge, there is no empirical evidence suggesting that these variables might play an important role in interval timing abilities.

The fact that some of the depressive patients were under medication might have attenuated the effects of depression on time perception, although none of the previous studies on this topic differentiated between patients on versus off medication. To gain insight into potential effects of medication, we conducted additional analyses of all dependent variables, using repeated-measures analyses including the between-subjects factor group with three levels: controls (*N* = 22), depressed patients under medication (*N* = 7), and depressed patients without medication (*N* = 15). A mixed-model approach was used that is valid for the resulting different group sizes (Kowalchuk et al., 2004). For none of the dependent variables, the effect of group or the interaction of group with any of the within-subjects factors was significant. Thus, the medication status does not appear to represent an important moderator variable.

How could the discrepancy between previously reported clear effects of depression especially on subjective time experience on the one hand, and a lack of effects on interval timing and time experience in our controlled experimental tasks on the other hand be explained (cf. Droit-Volet, 2013)? We conjecture that this difference is caused by the different contexts associated with the two types of tasks. If a depressive patient is asked to describe the passage of time, then he might imagine himself sitting at home, doing nothing, and experiencing a depressed mood and negative thoughts. It would be no surprise if in such a situation the passage of time were experienced as painfully slow, simply because the subject is not actively engaged in a task, and because he or she is in a depressive state. Additionally, revisiting our discussion of effects of attention on time perception, it could be argued that a depressive subject pays more attention to the mere passage of time than a healthy person does, for instance simply because depressive subjects are less active (Kitamura and Kumar, 1983; Münzel et al., 1988). In contrast, in the laboratory situation, the subject is actively engaged in a simple but demanding task, and has to concentrate on this task in order to achieve good performance. Thus, the subject is not inactive but active, and on rare occasions might even experience flow (Csikszentmihalyi, 1990). Additionally, the laboratory task is likely to draw attention away from the own self, thus attenuating the depressive state (Münzel et al., 1988). It would be interesting to investigate these ideas in more detail, by including measures reflecting the current emotional state or cognitive state of the subjects. It should also be taken into consideration that depressive patients typically show impairments in their attentional capacities. Such capacities are certainly needed when judging longer intervals in the range of several seconds to minutes (e.g., Grondin, 2010, 2012; Bangert et al., 2011; Gil and Droit-Volet, 2012). Thus, reduced attention is not able to explain the absence of effects of depression in our data, it may, however, be at the heart of previous findings that found underestimation of time. Taken together, the diverging results on the subjective experience of time on the one hand and time estimation and perception in controlled experimental tasks on the other hand, could be aligned by acknowledging the different situational demands. Alternatively, as discussed above the time frame considered when judging the flow of time might involve much longer intervals (e.g., hours or even days) than the durations studied in our experiment. In addition, depression might have different effects on retrospective compared to prospective time judgments (e.g., Tobin et al., 2010). In our study, the retrospective judgment of the time spent in the lab did not differ significantly between depressive patients and controls. However, it would be interesting to study effects of depression on this type of time judgments in greater detail.

In conclusion, our data do not provide evidence for a sizeable effect of depression on time perception, neither on the three classical prospective timing tasks (verbal estimation, production, reproduction), nor on the timed action task (TTC estimation), which we studied for the first time in depressive patients, nor for a retrospective time judgment.

## Conflict of Interest Statement

The authors declare that the research was conducted in the absence of any commercial or financial relationships that could be construed as a potential conflict of interest.
